# The gut microbiota, environmental factors, and links to the development of food allergy

**DOI:** 10.1186/s12948-020-00120-x

**Published:** 2020-04-02

**Authors:** Khui Hung Lee, Yong Song, Weidong Wu, Kan Yu, Guicheng Zhang

**Affiliations:** 1 School of Public Health, Curtin University of Technology, Bentley, WA Australia; 2 Curtin Health and Innovation Research Institute, Curtin University, Perth, WA 6102 Australia; 3 School of Public Health, Xinxiang Medical University, 601 Jinsui street, Xinxiang, Henan China; 4 School of Science, Edith Cowan University, Joondalup, WA Australia

**Keywords:** Environmental factors, Food allergy, Immune system, Microbiota, Probiotics

## Abstract

Food allergy appears to have its roots in an insufficient exposure to a diverse range of environmental microbiota during early life. Microbial exposure ensures the colonization of the gastrointestinal tract with commensal microbes, which is necessary for the induction of a balanced and tolerogenic immune function. High-throughput sequencing technology has facilitated in-depth studies of the gut microbiota as well as bacterial-derived metabolites. Although the role of the microbiota in allergies is now widely studied, its importance for food allergy was only recently noted. Studies in human cohorts have shown that there is an association of dysbiosis and pathogenesis of food allergy, while studies from animal models have demonstrated the capacity of specific species in the gut microbiota to alter immune response, which may lead to the desensitization of food allergy. This article reviews the role of the gut microbiota in food allergy, and discusses the influence of environmental factors as well as prevention and management strategies relating to such regulatory mechanism.

## Introduction

For decades, many cultivated microorganisms have been discovered by the microbiological culture technique, but these represent only a minority of the microbial species of the gut. The use of next generation high-throughput sequencing techniques has widened tremendously our knowledge of the human microbiota composition and its relationship to disease. The human body is estimated to consist of 10–100 trillion microbes, and more than 1000 bacterial species [1]. The highest number of microbes are found in the human gut [2]. The number of microbes found in the human gut are 10 times the number of cells making up the human body [3], although this ratio has been disputed [4]. Studies in the past few years have identified a critical role of the gut microbiota in shaping the immune system [5–8]. The gut microbiota is involved in the development of the organs of the immune system, and determines the tendencies of host immune responses. Research on the association between immune diseases and gut microbiota has reported that alterations in commensal bacteria can induce changes to the immune system [2, 9] which affect regulation of host metabolism, immune system maturation, and development of oral tolerance [10]. Thus, attempts to mitigate allergic diseases through adjusting the diversity and individuality of the gut microbiota have increased.

This review article outlines recent insights into the role of the gut microbiota in food allergy, the influence of environmental factors as well as prevention and management strategies involving the gut microbiota.

## Dysbiosis and food allergy

Dysbiosis refers to a change in the microbiota composition and function such that it disrupts gut homeostasis and contributes to diseases [11]. There are increasing evidence from human studies suggesting that dysbiosis is associated with pathogenesis of food allergy (Table 1) [12–23]. Although these studies are not able to identify specific bacterial taxa that are consistently associated with food allergy due to heterogeneity in study design, such as different sampling time points, different techniques used to characterize the gut microbiota, and different allergic phenotypes, these studies show microbiota diversity and composition are significantly associated with the onset of food allergy.

**Table 1:** Main gut microbiota differences between patients with and without food allergy

Study	Types of food allergy	Association with food allergy	References
Bunyavanich et al.	Cow’s milk	↓Clostridia, Firmicutes	[12]
Savage et al.	Cow’s milk, egg, wheat, soy, nuts	↓*Citrobacter*, *Oscillospira*, *Lactococcus*, *Dorea*	[14]
Azad et al.	Cow’s milk, egg, peanut	↑*Enterobacteriaceae* ↓*Bacteroidaceae*	[15]
Hua et al.	Peanut	↓Clostridiales ↑Bacteroidales	[16]
Inoue et al.	Egg, wheat, soybean, sesame, cow’s milk, peanut, shrimp, crab	↓*Dorea*, *Akkermansia* ↑*Veillonella*	[17]
Ling et al.	Cow’s milk, egg, wheat, nut, peanuts, fish, shrimp, soy beans	↓Bacteroidetes, Proteobacteria, Actinobacteria ↑Firmicutes	[18]
Chen et al.	Egg white, cow’s milk, wheat, peanut, soy bean	↓Bacteroidetes ↑Firmicutes	[19]
Fazlollahi et al.	Egg	↑*Lachnospiraceae*, *Streptococcaceae*, *Leuconostocaceae*	[20]
Dong et al.	Cow’s milk	↑Lactobacillaceae ↓*Bifidobacteriaceae*, *Ruminococcaceae*	[115]
Diaz et al.	Cow’s milk	↓*Coriobacteriaceae*	[21]
Berni Carnani et al.	Cow’s milk	↑*Bacteroides*, *Alistipes*	[22]
Kourosh et al.	Tree nuts, fish, milk, egg, sesame, soy	↑*Oscillibacter valericigenes*, *Lachnoclostridium bolteae*, *Faecalibacterium* sp.	[23]

Until now, it remains unclear how dysbiosis exactly affects the immune system in the development of food allergy but studies suggest that the gut microbiota influences the immune system by affecting host metabolism [2, 24–26] and the alteration of adaptive immunity [27, 28].

## Metabolic and immune effects

Major attention has been directed to the possible role of short-chain fatty acids, such as butyrate, propionate, and acetate in affecting the immune system (Table 2) [29–32], since short-chain fatty acids are the main product of the digestive action of the gut microbiota [29, 30]. Production of short-chain fatty acids, particularly butyrate, is able to enhance the Vitamin A metabolism, in turns inducing the activity of ALDH in CD103+MLN DCs, and increasing the percentages of T regulatory (Tregs) cells and increasing IgA production [33]. Meanwhile, short-chain fatty acids are able to inhibit histone deacetylases activity, resulting in regulation of *aldh1a1* expression, which contributes to immune tolerance. Other than this, short-chain fatty acids can bind metabolite-sensing G-protein coupled receptors, GPR43 or GPR109A [34], in turn promoting the tolerogenic CD103+ DC function and protecting against food allergies [35]. Moreover, short-chain fatty acids can reduce the production of pro-inflammatory cytokines including IL-1β, IL-6, IL-17 [36], and meanwhile increase the production of anti-inflammatory mediators including IL-10 [36, 37]. Thus, short-chain fatty acids are viewed as a key factor in promoting immunological tolerance towards harmless antigen and preventing inflammation.

**Table 2:** Production of the short-chain fatty acids by the gut microbiota

Short-chain fatty acids	Pathway	Microbiota	References
Acetate	Acetyl-CoA	Most enteric bacteria	[31]
	Wood–Ljungdahl pathway	*Blautia hydrogenotrophica*	
Propionate	Succinate pathway	Negativicutes, Bacteroidetes	[32]
	Acrylate pathway	*Megasphaera elsdenii*, *Clostridium propionicum*	
	Propanodiol pathway	*Salmonella enterica*, *Roseburia inulinivorans*, *Ruminococcus obeum*	
Butyrate	Butyrate kinase and phosphotransbutyrylase	*Coprococcus* species	[31]
	Butyryl-coenzyme A (CoA):acetate CoA-transferase	*Faecalibacterium prausnitzii*, *Roseburia* spp., *Eubacterium rectale*, *Eubacterium hallii Anaerostipes* spp.	

## Adaptive immunity

Adaptive immune responses are divided into two types: humoral immunity, regulated by B cells [38, 39], and cell-mediated immunity, regulated by T cells. The role of Tregs, subset of CD4+ T cells in oral tolerance development to food allergen, have been confirmed in animal models [40, 41] as well as human studies [42, 43] in which the induction of allergen-specific Treg cells is highly associated with a favourable allergy outcome. Microbiota, especially Clostridia species, in this case, were shown to be able to induce the production of Tregs [44, 45], which helps to inhibit allergic inflammation and promote oral tolerance [12, 46–48].

## Specific Taxa related to food allergy across multiple studies

Bacteroidetes and Firmicutes comprise 90% of the microbiota population [49] and the colonization of these two microbiota phyla is associated with the pathogenesis of food allergy [50], as summarized in Table 3.

**Table 3:** Gut microbiota taxa and alterations associated with food allergy

Phylum	Class	Genus	Species	Action in the Gut	References
Bacteroidetes	*Bacteroidetes*	*Bacteroidetes*	*Bacteroides fragilis*	Increase the suppressive capacity of Tregs and induce the production of IL-10 from Foxp3+ T cells	[51]
Firmicutes	*Clostridia*	*Clostridium* *Lactobacillus* *Ruminococcus*		Promote the accumulation of Tregs	[116]

## Bacteroidetes

An association between Bacteroidetes phyla in the gut and food allergy was found in a few epidemiologic studies [15, 18, 19]. Using gene sequencing methods, these studies identified that children with various types of food allergy had a lower relative abundance of Bacteroidetes compared with healthy children. The exact role of Bacteroidetes is unclear. However, a murine study indicated that *Bacteroides fragilis*, a species of Bacteroidetes, was able to produce polysaccharide A (PSA), which increased the suppressive capacity of the Treg cells and increased the production of IL-10 from Foxp3+ T cells [51]. The accumulated IL-10 mediates tolerance at mucosal surfaces and prevents intestinal inflammation. Further clinical studies in humans are needed to examine the regulatory role of Bacteroidetes in the development of food allergy.

## Firmicutes

Firmicutes is the largest microbiota phylum containing more than 200 genera [52]. Most of the Firmicutes detected in the gut belong to the *Clostridium* clusters *XIVa* and *IV*. Both groups comprise members of the genera *Clostridium*, *Eubacterium* and *Ruminococcus*. Firmicutes are the most important butyrate producer in the gut and help to reduce inflammation through the inhibition of histone deacetylase [30]. A number of studies using 16S rRNA gene sequencing have identified a lower relative abundance of bacterial class *Clostridia* (bacterial phylum Firmicutes) in children with food allergy compared to healthy children [12, 13, 53, 54]. It is an accepted scientific knowledge that *Clostridia* promotes the accumulation of Treg cells [44, 45, 47, 55]. However, a study conducted by Stefka st al [55] demonstrated that Clostridia elevated IL-22 expression by RAR-related orphan receptor gamma (RORγt)+ ILCs and T cells, which in turn increasing an early, innate, barrier protective response. At the same time, Clostridia also elevated the production of IL-17, which in turn reducing intestinal epithelial permeability. Contradictory results also exist that patients with food allergy showed an increase in levels of *Clostridia* [18, 19, 56]. The genera of Clostridia that have been identified so far to have an association with food allergy are *Clostridium* [13, 18, 19, 53] and *Ruminococcus* [13, 15, 19, 54]. Diesner et al. reported that mice tolerant to food allergy have higher abundances of Ruminococcaceae [54] compared with intolerant counterparts, supporting the potential protective effects of *Ruminococcus* on food allergy. Nevertheless some discordant results the Clostridia class is that more clearly involved in mechanism of food tolerance induction and in food allergy protection.

## Early-life colonization and environmental factors

Some studies argue that the gut microbiota is critical in early life for the development of the immune system as well as the development of allergic diseases [15, 18, 19, 53, 57]. The gut microbiota is unstable in the first 2–3 years of life during the initial colonization and development of the gut. Disruptions of microbial colonization during this period have been shown to increase the disease susceptibility during life. After the age of three the gut microbiota progresses towards an adult-like configuration and it essentially remains stable unless perturbed [1, 5, 53].

**Fig. 1:**
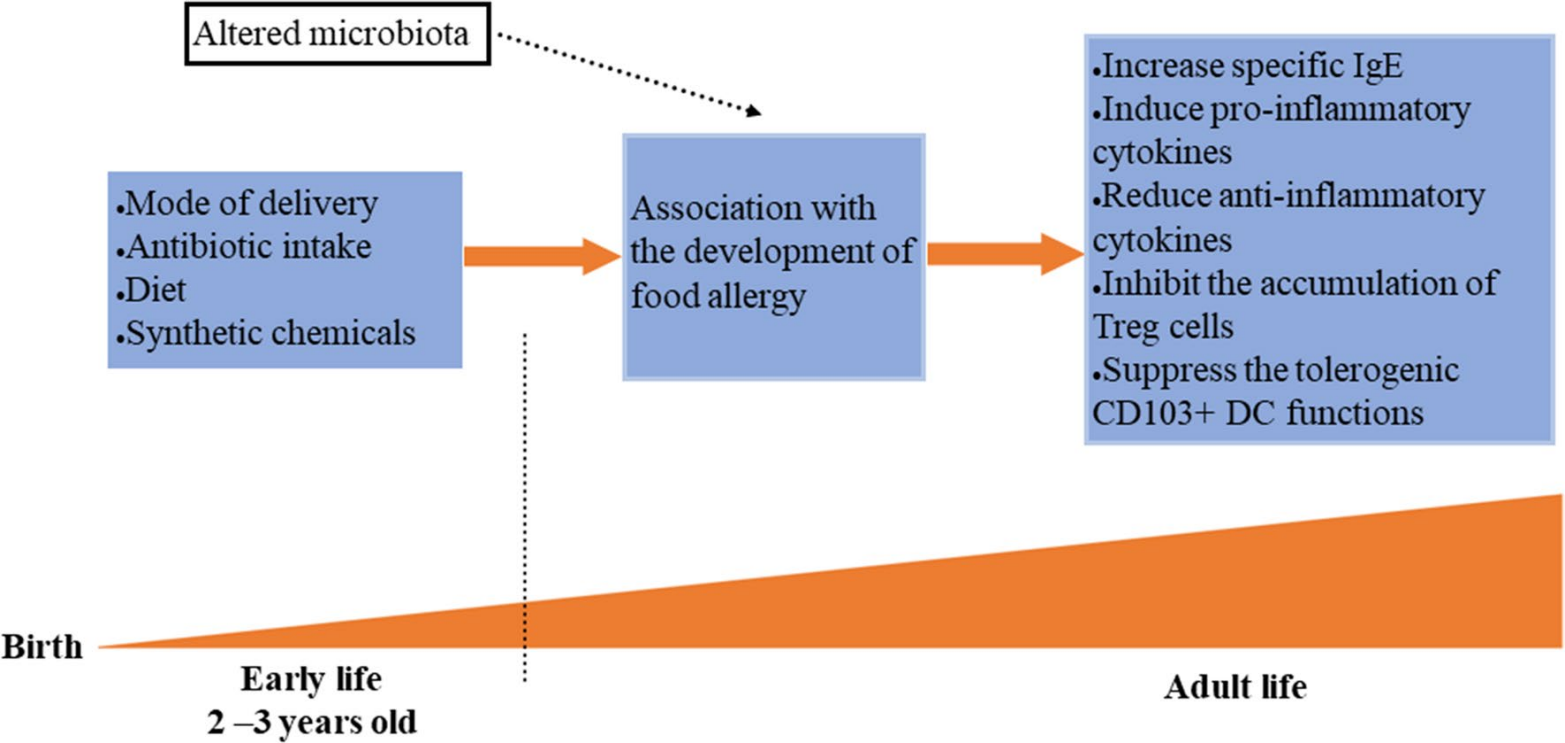
Early-life colonization and environmental factors. The gut microbiota is unstable in the first 2–3 years of life during the initial colonization and development of the gut. During this period, the gut microbiota is vulnerable to some disruptions such as mode of delivery, antibiotic intake, diet and synthetic chemicals, which may increase specific IgE, induce pro-inflammatory cytokines, reduce anti-inflammatory cytokines, inhibit the accumulation of Treg cells, and suppress the tolerogenic CD103+ DC (dendritic cell) functions, contributing to the pathogenesis of food allergy. After the age of 3 the gut microbiota progresses towards an adult-like configuration and this remains the same throughout the life

The dramatic rise of food allergy in modern society has led to the postulation of the hygiene hypothesis [58]. The hygiene hypothesis proposes that a lack of early childhood exposure to infectious agents suppresses the development of the immune system which leads to the rise of atopic diseases. Recent work has revisited the hygiene hypothesis model to include mode of delivery, antibiotic intake, diet [59] and synthetic chemicals as factors in altering gut microbiota (Fig. 1).

## Mode of delivery

Perturbation of gut microbiota may influence susceptibility to food allergy [60]. It is evidenced that infants born vaginally are exposed to both maternal fecal and vaginal microbiota while infants born by caesarean section are exposed to maternal skin and environmental microbes only [50]. For instance, infants born by caesarean section are more likely to have lower abundance of Bacteroides and higher abundance of Firmicutes. Both Bacteroides and Firmicutes were revealed to associate with development of food allergy [50]. A population based birth cohort study revealed that children born by caesarean section had a threefold higher chance of developing food allergy [61]. However, the role of the delivery mode in influencing the food allergy outcome remains unclear as the delivery mode only exhibits an effect on the immune system for the first 90 days [62].

## Antibiotic intake

Mothers undergoing a caesarean section are more likely to receive antibiotics [62], which can perturb the composition of the gut microbiota and modify the risk for allergic outcomes [63].

A number of studies have attempted to elucidate how antibiotics modify the composition of the gut microbiota and lead to the pathogenies of food allergy [64–66]. These studies revealed that antibiotic administration altered and reduced the microbiota diversity. A study suggested that antibiotic administration may cause reconstitution of the flora, thereby resulting in failure of the signal transmission via Toll-like receptor 4 (TLR4) [64]. The inability to signal via TLR4 resulted in markedly increased peanut-specific IgE and Th2 cytokine responses, contributing to allergic responses. Nevertheless, the association of antibiotic intake in early life and the development of food allergy is debated [67]. In a Chicago Family Cohort Food Allergy study, 1359 families were recruited and structure questionnaires were administered. This study did not find any association between antibiotic intake in the first year of life and the diagnosis of food allergy [67].

## Diet

Diet plays an important role in the establishment of the gut microbiota and affects allergic symptoms [68, 69]. The dietary intake of infants starts with milk, either breast milk or formula [70]. Breast milk contains human milk oligosaccharides which stimulate the growth of *Bifidobacterium bifidum* [71] and genus *Lactobacillus* [72], which are the main probiotic organisms in the gut, forming an acidic environment with enriched short-chain fatty acids [73]. Consequently, breastfed infants are colonized with *Bifidobacteria* and *Lactobacilli* compared with formula fed infants [74]. Additionally, the breastfeeding duration also has an influence on the development of food allergy as infants with a very brief breastfeeding duration have a higher risk of developing cow’s milk allergy [75].

The milk intake is gradually replaced by a solid food diet that is similar to that of the adult and composed of a wide-variety of dietary macronutrients such as proteins, fats, carbohydrates and fibres [76]. These macronutrients are fundamental in determining microbiota composition and its effect on health outcomes.

After the introduction of complementary diet, the gut of infants exhibit more *Lactobacillus*, *Ruminococcus*, *Bacteroides*, *Peptostreptococcus* and *Clostridium*, which are tryptophan-catabolizing species [77]. Tryptophan is an amino acid commonly presented in high protein food, which is required for protein biosynthesis. Tryptophan metabolism plays a fundamental role in regulating the immune response as well as T cell proliferation. Other than this, tryptophan metabolism also induces IL-10 receptor-1 (IL-10R1) expression, which is essential in determining whether cells respond to IL-10, a potent anti-inflammatory cytokine that inhibits the release of pro-inflammatory cytokines [78].

Other than protein, fibre can also affect microbiota composition. The microbiota composition of the human gut associated with a high fibre diet is different from that seen with a high fat diet [79]. The gut microbiota with a high fat diet consisting of animal-based foods has higher abundance of *Alistipes*, *Bilophila* and *Bacteroides* and lower abundance of *Roseburia*, *Eubacterium* and *Ruminococcus* at genus level when compared to the gut microbiota with a high fibre diet [79]. The microbial conversions of dietary fibre to monosaccharides involve a number of metabolic pathways catalysed by the enzymatic activities of the gut microbiota and short-chain fatty acids are the major end-products of these microbial conversions [29, 30]. Mice studies were conducted in order to investigate the role of dietary fibre in suppressing immune response to food antigens [35, 80]. In these studies, mice were sensitized with food antigen and fed with either high fibre diet or no fibre diet. After few weeks of exclusion diet, mice receiving high fibre diet, showed significantly reduced symptoms of food allergy. Therefore, the “Western” diet with its high fat but low fibre content is proposed to be one of the reasons for the high prevalence of food allergy in Western countries [35, 81].

## Synthetic chemicals

Synthetic chemicals play a role in perturbing the composition of gut microbiota as microbial metabolism of chemicals by gut microbiota may cause microbial dysbiosis [82], which in turn triggers allergy reaction [83, 84]. For example, food additives, one of the synthetic chemicals, are commonly found and used in our food as antioxidants, colourings, and flavourings, sweeteners and preservatives [83]. Mouse studies have indicated that such chemicals are associated with increased risks for food allergy [83, 84]. Food additives inhibited the accumulation of Treg cells, which is necessary for acquisition of oral tolerance [83]. The food additives also promoted the induction of allergic CD11b+ DCs, reduced the accumulation of tolerogenic CD103+ DCs as well as inhibited the induction of CD4+CD25hiT cells. Further to this, this study also suggested that intake of multiple food additives could increase the risk of developing food allergy.

Other synthetic chemicals, such as air pollutants, can be responsible for increasing the appearance of food allergy. Higher ambient levels of air pollutants, especially nitrogen dioxide have been consistently demonstrated to be associated with the increasing risk of allergies, including food allergies [85–87]. The mechanism of how air pollutant can cause food allergy remains unknown but studies suggested that inhaled air pollutants were able to directly or indirectly modify the composition of gut microbiota [88], which may cause an increase in the gut permeability and inflammation. Furthermore, inhaled air pollutants can lead to a substantial inflammatory response via reactive oxygen species (ROS) production and nuclear factor kappa B (NF-κB) activation in GI tract.

## Therapeutic strategies

Human [12, 15, 18, 19, 53] and murine [54] models have demonstrated that study subjects with food allergy have distinct gut signatures and a different gut microbiota composition. Moreover, there is growing evidence that alteration of the gut microbiota may explain the development of food allergy [40, 41]. Microbial colonization has been shown to promote the induction of Treg cells, which is necessary to modulate the immune system and maintain tolerance to self-antigens [40, 41]. Microbiota may also influence the epigenetic modification of genes. It has been demonstrated that various forms of epigenetic changes, such as DNA methylation and histone modification, regulate the immune system. Also microbial derived metabolites, such as short-chain fatty acids, have been shown to reduce pro-inflammatory cytokines and induce anti-inflammatory mediators as well as inhibit histone deacetylases [36].

Research indicates that environmental factors affect the microbiota composition making it an ideal target of research to find new interventions to desensitize food allergy.

## Probiotic supplementation

The use of probiotic supplementation seems an attractive option for the prevention and treatment of allergic diseases. Probiotics are defined as “live microorganisms which, when administered in adequate amounts as part of food, confer a health benefit on the host” [89, 90]. Probiotics can act as promoters of an adequate balance in the gut microbiota to prevent the development of allergies. The beneficial effects of probiotics involve restoring intestinal permeability to normal, improving the intestine’s immunological barrier function (both physical and mucous layer), promoting IgA production and inhibiting the release of proinflammatory cytokines through regulating gut microbiota composition [91, 92].

A well-characterised bacterial probiotic in desensitizing food allergy, especially cow’s milk allergy, is *Lactobacillus rhamnosus* GG [93]. Dietary intervention with *Lactobacillus rhamnosus* GG was found to reduce allergic responses towards cow’s milk in murine [94, 95] and human studies [93, 96, 97]. *Lactobacillus rhamnosus* GG was able to increase the production of different cytokines with proinflammatory (*TNF*-*α* and *IL*-*6*) or regulatory *(IL*-*10*) functions [98]. In addition, *Lactobacillus rhamnosus* GG also induced the accumulation of colonic Treg cells in the intestine [99]. In addition, *Lactobacillus rhamnosus* GG was able to increase *FoxP3* demethylation rate, increase IL-4 and IL-5 DNA methylation rate, reduce IL-10 and IFN-γ DNA methylation rate, increase the expression of miR-155, -146a, -128 and -193a, to promote the acquisition of cow’s milk tolerance 12 months after treated with extensively hydrolyzed casein formula containing the probiotic *Lactobacillus rhamnosus* GG [97].

One study found that *Lactobacillus rhamnosus* GG showed a weak effect in desensitizing peanut allergy [100]. Most of the participants who received this probiotic and peanut oral immunotherapy passed the oral food challenge after receiving the treatment for 18 months and still passed the oral food challenge after 2 to 5 weeks after that. Moreover, participants who received probiotic and peanut oral immunotherapy showed a decrease in peanut sIgE levels and skin prick test wheal size, as well as an increase in peanut sIgG4 levels, at the end of treatment. Three months after treatment ended, these participants still had low sIgE levels and a small skin prick test wheal size. However, there was no convincing evidence that probiotic was effective in reducing allergic reactions to food as this study only compared between participants who received probiotic and peanut oral immunotherapy and participants who received placebo without including oral immunotherapy group only and probiotic group only. Also the prolonged tolerance towards peanut remains unclear as the participants in this study were not followed up beyond 3 months.

A mouse model of shellfish allergy demonstrated that oral administration of the probiotic strain *Bifidobacterium longum* reduced the specific IgE and stimulated dendritic cell maturation and CD103+ tolerogenic DCs accumulation in gut-associated lymphoid tissue. This, in turn, increased Tregs differentiation and suppressed Th2 responses [95, 101]. A murine study of cow’s milk allergy showed that oral administration of *Bifidobacterium longum* subsp. *Infantis* LA308 strain induced the expression of IL-10 and skewed the immune response to Th1. These results are encouraging to find a candidate probiotic strain to perform a clinical trial.

Two other murine studies have reported the capacity of *Clostridium butyricum* to reduce adverse reactions to egg and cow’s milk [46, 102]. The mechanism of how *Clostridium butyricum* is able to inhibit allergic inflammation remains unknown. Shi [102] suggested that the therapeutic effect of *Clostridium butyricum* may be generated by IL-10, as the IL-10-producing antigen specific Breg was found in mice with egg allergy which were administered with specific immunotherapy and *Clostridium butyricum*. On the other hand Zhang [46] suggested that *Clostridium butyricum* increased sIgA, CD4+CD25+Foxp3 Treg cell as well as reversed the imbalance of Th1/Th2 and Th17/Treg. These interesting results require further validation from human clinical trials.

There are three limitations on probiotic dietary intervention. First, the effect of probiotic is strain specific [103] and may vary depending on the individual’s lifestyle and baseline gut microbiota profile [104]. Secondly, abrupt termination of probiotic dietary intervention may further increase the gut dysbiosis [105]. Lastly, there are only a few studies about probiotic dietary intervention and further studies are needed to validate the findings in human system.

## Prebiotic supplementation

Prebiotic is defined as “nonviable food component that confers a health benefit on the host through modulation of the gut microbiota” [106]. An animal study reported that cocoa, a source of antioxidant polyphenols, may be used to desensitize oral allergy [107]. In this study, rats administered with a 10% cocoa diet achieved oral tolerance and had a lower relative abundance of bacterial phylum Firmicutes and Proteobacteria and a higher relative abundance of Tenericutes and *Cyanobacteria* spp. compared to rats who received a standard diet, either orally sensitized or non-sensitized rats. It was suggested that the cocoa diet was able to increase the proportion of TCRγδ+ and CD103+CD8+ cells and decrease the proportion of CD62L+CD4+ and CD62L+CD8+ cells in mesenteric lymph nodes [108], regulate Treg cells function and reduce IgA production [107] through modulation of gut microbiota. This suggests that a cocoa diet has potential in regulating allergic immune responses in the human body and is of interest to investigate further.

## Synbiotic supplementation

Synbiotics refers to a mixture of probiotics and prebiotics, designed to improve the survival of the beneficial microbiota as well as stimulate the growth of beneficial microbiota in the gastrointestinal tract [106].

A pioneer study tested a prebiotic blend of fructo-oligosaccharides with the probiotic strain Bifidobacterium breve M-16V. Infants with suspected non-IGE cow’s milk allergy were administered with a hypoallergenic, nutritionally complete amino-acid based formula either with or without the synbiotics [109]. The relative abundance of *Bifidobacteria* was increased in infants with cow’s milk allergy who were administered synbiotics. Moreover, the microbiota profile of these allergic infants became similar to that of healthy breastfed infants. More studies are required to confirm this therapeutic effect of synbiotics supplementation in desensitizing food allergy. It is a promising therapeutic strategy for improving the gut ecosystem and reducing food allergy responses.

## Fecal microbiota transplantation

Fecal microbiota transplantation is a possible therapeutic for food allergy. Transplantation of fecal bacteria from a healthy donor to a disease recipient can re-establish gut microbiota diversity leading to the resolution of symptoms [110, 111]. As dysbiosis affects the development of food allergy [12], restoration of immune homeostasis and reconstruction of the impaired gut microbiota barrier by fecal microbiota transplantation may be able to promote the development of oral tolerance [110]. Recently, a human study has revealed that fecal microbiota transplantation is able to induce remission of infantile allergic colitis through restoration of gut microbiota diversity [112]. Although the available data in this field remain limited and the relevant scientific work has only just begun, this recent success in reducing infantile allergy colitis symptoms suggests that fecal microbiota transplantation can be a feasible strategy to arrest food allergy responses.

## Synthetic stool substitute

Considering the strain specific effect of probiotic [103] and limited patient acceptance of fecal microbiota transplantation [113], a synthetic stool substitute was proposed in a pilot study [113]. In this study, a stool substitute with 33 strains was developed based on the fecal microbiota diversity of a healthy donor, also known as RePOOPulate. *Clostridium difficile* infection symptoms of patients were eradicated after 2–3 days of RePOOPulate treatment and this symptom free state lasted for 6 months. This synthetic stool substitute is so far only tested on two patients with *Clostridium difficile* infection, yet its potential benefit of reverting normal bowel pattern and restoring immune homeostasis may help to reduce allergic reactions towards food.

## Microbiome-based therapy

Microbiome-based therapy can be viewed as a potential way in treating food allergy. In a mouse study, germ free mice were colonized with human feces from infants with cow’s milk allergy and age and gender matched healthy infants [114]. Healthy-colonized mice had a higher abundance of *Anaerostipes caccae* when compared with cow’s milk allergy colonized mice. Mice were then administered with *Anaerostipes caccae* in order to further investigate the role of *Anaerostipes caccae* in regulation of gene expression. *Anaerostipes caccae* were able to reduce the expression of Th2 dependent, antibody (serum BLG-specific IgE and IgG1), *Acot12* expression as well as cytokine responses IL-13 and IL-4, which promoted oral tolerance towards cow’s milk allergy. This opens up a new perspective of food allergy therapy on human.

## Conclusion

Studies have shown that the gut microbiota composition is vulnerable to disruptions in early life and that associated changes in host-microbiota homeostasis can cause food allergy. These studies support a regulatory role of the gut microbiota in the manifestation of food allergy, particularly in early life, but many questions remain and the underlying mechanisms are yet to be defined. The majority of our knowledge stems from animal studies and more human studies are required to validate the precise role of the gut microbiota in the development of food allergy. Evidence is emerging to suggest that therapeutic strategies in modifying gut microbiota composition are useful in the prevention, management and treatment of food allergy. Such strategies are apparent future research directions for developing prophylactic and therapeutic approaches against food allergy.

## Data Availability

Not applicable.
